# Spacing Repetitions Over Long Timescales: A Review and a Reconsolidation Explanation

**DOI:** 10.3389/fpsyg.2017.00962

**Published:** 2017-06-20

**Authors:** Christopher D. Smith, Damian Scarf

**Affiliations:** Department of Psychology, University of OtagoDunedin, New Zealand

**Keywords:** spacing effect, distributed practice, reconsolidation, learning, retention, inverted-U curve

## Abstract

Recent accounts of the spacing effect have proposed molecular explanations that explain spacing over short, but not long timescales. In the first half of this paper, we review research on the spacing effect that has employed spaces of 24 h or more across skill-related tasks, language-related tasks and generalization for adults and children. Throughout this review, we distinguish between learning and retention by defining learning (or acquisition) as performance at the end of training and retention as performance after a delay period. Using this distinction, we find age- and task-related differences in the manifestation of the spacing effect over long timescales. In the second half of this paper, we discuss a reconsolidation account of the spacing effect. In particular, we review the evidence that suggests the spacing of repetitions influences the subsequent consolidation and reconsolidation processes; we explain how a reconsolidation account may explain the findings for learning; the inverted-U curve for retention; and compare the reconsolidation account with previous consolidation accounts of the spacing effect.

## Introduction

The *spacing effect* is the observation that repetitions spaced in time tend to produce stronger memories than repetitions massed closer together in time. Research on the spacing effect dates back to Ebbinghaus (1885) and his book*, Memory: A Contribution to Experimental Psychology*. Despite the great deal of research that has been conducted on the spacing effect since Ebbinghaus, a consensus is yet to be reached regarding the mechanism(s) underlying the effect. Part of the difficulty in developing a consistent mechanistic account is that the spacing effect occurs under a wide variety of conditions. Some researchers have dealt with this difficulty by proposing dual process models wherein different mechanisms explain the spacing effect in different tasks (e.g., Greene, 1989). In contrast, we along with other researchers (e.g., Estes, 1955; Hintzman, 1974; Naqib et al., 2012; Smolen et al., 2016), suggest that the spacing effect may involve different mechanisms depending on the *duration* of the spacing interval. In the first half of this paper, we review behavioral studies that explore the effects of spacing intervals of 24 h or more on skill-related tasks, language-related tasks and the generalization of learning. We excluded studies outside of these categories because they do not give any further insight into the spacing effect over long timescales beyond demonstrating that spacing is also beneficial for other types of material such as physics (Grote, 1995) and mathematics (Rohrer and Taylor, 2007). Additionally, based on the methods and results of studies using intervals of 24 h or more, there was a natural division between children and adults, and this is reflected in the first half of the current paper.

In the second half of this paper, we propose a memory reconsolidation account of the spacing effect that can explain the results of the experiments covered in the first half. Our theoretical approach is complementary to recent reviews that discuss molecular mechanisms contributing to the spacing effect over timescales of seconds to several hours, but cannot explain the spacing effect over longer timescales (Naqib et al., 2012; Smolen et al., 2016). However, the reconsolidation account is also compatible with the idea that other mechanisms such as encoding variability or retrieval difficulty can explain the spacing effect over short to intermediate timescales. Additionally, throughout this paper, we generally define learning (or acquisition) as performance at the end of training or on an immediate test and retention as performance after a delay period. It is worthwhile to make this distinction because these two measures represent different aspects of task performance. Learning reflects the peak performance obtained in a task whereas retention reflects the rate of forgetting.

## The Spacing Effect in Skill-Related Tasks

### Adults

Studies utilizing skill learning tasks to investigate the spacing effect have, by and large, compared a group that completes all of its training within 1 day (i.e., massed) to a group that completes its training across multiple days (i.e., spaced). As one might expect, spaced practice generally leads to better learning and retention than massed practice (Shea et al., 2000; Dail and Christina, 2004; Arthur Jr. et al., 2010). Spacing has proven beneficial for a wide range of skills such as playing video games (Shebilske et al., 1999; Stafford and Dewar, 2014), interviewing (Heidt et al., 2016), learning surgical skills (Verdaasdonk et al., 2007; Spruit et al., 2014; Andersen et al., 2016), playing a piano sequence (Rubin-Rabson, 1940; Simmons, 2011), balancing on a swaying platform (Shea et al., 2000), electrical testing of a vehicle charger (Hagman, 1980), learning to enhance alpha waves through bio-feedback (Albert et al., 1974) and golf putting (Dail and Christina, 2004).

#### Intensity of Training

Most of the studies that investigate the spacing effect for skill-related tasks compare a group that completes their sessions across multiple days to one that completes all sessions within a single day. A smaller number of studies have investigated, when training is spaced over multiple days, whether manipulating the intensity of training influences learning and retention. For example, is 1 h of training per day for 16 days better than the more intense 4 h of training per day for 4 days? Baddeley and Longman (1978) addressed this question by varying the number of sessions per day (1 or 2) and the number of hours in a session (1 or 2) that postmen were trained to type. After approximately 60 h of training, postmen who completed the least intense training (1 session of 1 h per day) learned to type faster and more accurately than the other, more intense groups. Additionally, a series of retention tests conducted 1, 3 and 9 months after training revealed a somewhat less clear-cut result, this was partly due to the least intense group going on holiday and completing less training than the other groups, where the overall trend was for the group with the most intense training (2 sessions of 2 h per day) to perform worse than the other groups. There are some other studies, though generally less systematic, that are consistent with Baddeley and Longman’s (1978) finding that less intense training, spread across a larger number days, provides better learning (Lashley, 1915; Ruch, 1928; Knapp and Dixon, 1950; Knapp et al., 1958; Kauffeld and Lehmann-Willenbrock, 2010; De Win et al., 2013).

Obviously, despite Baddeley and Longman’s (1978) finding that the least intense training resulted in the greatest learning, there probably are lower limits on training intensity after which performance declines. For example, Paik and Ritter (2015) had participants learn to balance an inverted pendulum under one of four different practice schedules. For our purposes the four practice schedules can be characterized from least intense to most intense. Paik and Ritter (2015) found that the intermediate intensity group (the hybrid-massed group), who completed 8 sessions across 1 week, showed significantly better learning than the least intense group (the spaced group), who completed 8 sessions across 2 weeks.

Another approach to investigating the intensity of daily sessions is by varying the total number of trials per day. Studies investigating perceptual and visuo-motor learning have found that a minimum number of trials is required for learning to occur (Wright and Sabin, 2007; Aberg et al., 2009), that there is an optimal number of trials per day for learning and going beyond this optimum produces minimal additional learning (Savion-Lemieux and Penhune, 2005; Goedert and Miller, 2008; Molloy et al., 2012). For example, Wright and Sabin (2007) had participants learn to either discriminate the frequency of tones or the time interval between tones, for 360 or 900 trials per day. For discriminating the frequency of tones, participants who received 360 trials per day failed to improve above their baseline level of performance, while participants in the 900 trials per day group displayed consistent improvement. In contrast, when discriminating the interval between tones, participants in both groups displayed the same rate of learning, indicating that going beyond 360 trials per day had no impact on this particular discrimination.

When learning a new sport or skill, many people will practice for just a few days per week. It is therefore theoretically and practically interesting to understand the spacing effect when learning occurs on a weekly basis. Young (1954) had college students learn and practice badminton or archery, for 2 or 4 days per week. For badminton, the students improved more when they practiced 2 days per week compared to 4 days per week. In contrast, the archery students improved more when they practiced 4 days per week compared to 2 days per week. Young (1954) speculated that the results were due to differences in participants’ prior experience of skills related to the two sports. Many participants probably had prior experience in racket sports and this meant they could improve their badminton skills with relatively spaced sessions, but for archery they required more concentrated sessions to build up their basic skills.

Harmon and Miller (1950) had college women who had no prior experience playing billiards learn and practice with different schedules, for a total of 9 sessions. They compared four different schedules: group 1 completed 3 sessions per week for 3 weeks, group 2 completed the 9 sessions across 9 consecutive days, group 3 completed sessions across a gradually increasing interval (i.e., they practiced Day 1, 2, 3, 5, 8, 13, 21, and 34), and group 4 completed 1 session per week for 9 weeks. At the end of training, group 3 performed significantly better than the other three groups. Somewhat similar to Young’s (1954) explanation noted above, Harmon and Miller (1950) attributed the better performance of group 3 to participants initially benefiting from concentrating their sessions to reach a certain threshold of learning and then benefiting from the spacing of sessions to further improve performance.

Finally, there are some studies which do not find a spacing effect. Some of these studies potentially reflect the fact that the tasks used are less sensitive to manipulations of training intensity because the training for all of the groups in these studies occurs over a long period of time and the tasks used are different to those reported on earlier (Franklin and Brozek, 1947; Massey, 1959; Murphree, 1971; Romkema et al., 2015). A few other studies potentially do not find a spacing effect because prior experience leads to a fast rate of learning and/or very little forgetting (Schendel and Hagman, 1982; Mitchell et al., 2011).

Overall, less intense daily training where learning is distributed over a larger number of days enhances learning and retention compared to more intense daily training. However, a certain minimum threshold of experience seems to be necessary for learning to occur in these daily sessions. This threshold varies depending on the type of task. Additionally, it would be useful to see whether the beneficial effect of gradually expanding the spacing interval found by Harmon and Miller (1950) could be replicated for billiards and other tasks to furnish theoretical accounts of the spacing effect and provide an effective schedule for learning and retaining skills.

#### Task Complexity

In their review of the spacing effect, Donovan and Radosevich (1999) found that for tasks categorized as highly complex (e.g., airplane control simulation) the effect size of the spacing effect was very small (*d =* 0.07). The implication of this finding is that it is not worthwhile to space the learning of complex tasks. Their analysis contained studies with intervals ranging from a few seconds to 24 h on a variety of tasks, so it is interesting to consider whether their findings apply to skill-related studies with spacing intervals of 24 h or more. Arthur Jr. et al. (2010) addressed this issue by training participants in a complex simulation game where participants played the role of the commander of a navy fleet. Participants completed their sessions spread across 2 weeks or concentrated in 1 week. The 2-week group displayed better learning on a post-test at the end of training than the 1-week group (*d* = 0.24) and better retention on a test 8 weeks after training (*d* = 0.46). It is important to note that Donovan and Radosevich’s (1999) massed groups completed their training all in a single day; if Arthur Jr. et al. (2010) had used a similar comparison group they probably would have reported even larger effect sizes.

Additionally, a number of studies have reported the benefits of spacing while learning surgical skills (Moulton et al., 2006; Verdaasdonk et al., 2007; Gallagher et al., 2012; De Win et al., 2013; Spruit et al., 2014; Kang et al., 2015). Some of these studies provide information on effect sizes. These studies generally find a medium to large effect size of spacing and therefore provide additional evidence that spaced repetitions produce a worthwhile improvement in complex skill tasks.

While the studies reviewed above suggest that it is worthwhile to space the learning of complex skills, this does not necessarily apply to other task domains. Indeed, a number of older studies assessing puzzle learning using spacing intervals of 1 day failed to find an advantage for spaced practice for learning (Cook, 1934; Garrett, 1940; Ericksen, 1942). For example, Garrett (1940) compared participants who learned a symbol-digit substitution task, a code-learning task or an artificial language task by spaced practice or massed practice. The spaced group completed 1 trial per day and the massed group completed all of their trials within a single day. Based on acquisition data, Garrett (1940) classified the symbol-digit substitution and code-learning tasks as less complex than the artificial language task. Fittingly, the symbol-digit substitution task and the code-learning task were acquired faster under spaced practice and the artificial language task was acquired faster under massed practice.

### Infants and Children

In addition to the literature on adults, there are also several studies that have explored the spacing effect for skill-related tasks in infants and children. For example, Vander Linde et al. (1985) compared infants who learned to kick to activate an overhead crib mobile with spaced or massed practice. The daily group completed 3 sessions across 3 consecutive days, the alternate-day group completed 3 sessions on alternate days and the massed group completed all 3 sessions on a single day. Consistent with the adult literature, infants in the alternate-day group learned the task significantly faster than infants in the daily and massed groups.

Studies have also investigated the spacing effect for retention in infants, utilizing the concept of a time window (Rovee-Collier, 1995). The time window is a limited period of time in which additional experiences can be integrated into a memory, beyond which the time window shuts because the memory has been forgotten. Rovee-Collier (1995) compares time windows to critical periods, in that time windows are a limited period of time in which an organism is responsive to certain experiences, except that the time window is psychological rather than biological. For example, in one study a new item was integrated into a pre-existing category if it occurred 4 days after the original category learning experience, but the new item was treated as a unique event if it occurred 5 or 6 days after category learning (Rovee-Collier, 1995). The time window concept uses the same basic principles to explain a range of phenomena in infant memory that involve integrating new experiences with related long-term memories (Rovee-Collier, 1995). Some of the phenomena explained using the time window concept are categorization, memory modification and the spacing effect (Rovee-Collier, 1995). For the spacing effect, studies using the time window concept have found that repetitions that occur later in the time window lead to a task being remembered longer than repetitions that occur earlier in the time window; however, if the repetition is outside the time window, even if it is only a single day, it is as if the infant is encountering it for the first time (Rovee-Collier et al., 1995; Hartshorn et al., 1998b; Hudson and Sheffield, 1998; Galluccio and Rovee-Collier, 2006).

**FIGURE 1:**
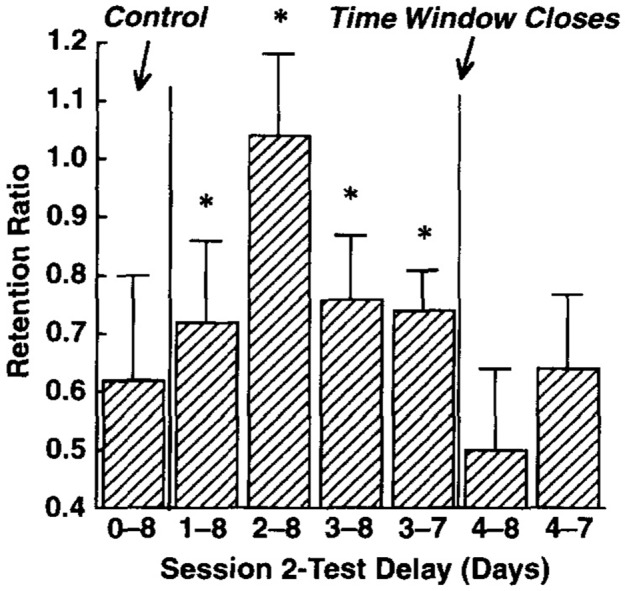
Infants’ retention performance after the memory was reactivated 1–4 days after training. Note that performance initially improves with spacing and then declines, forming an inverted-U relationship. Asterisks indicate whether performance was significantly above the baseline performance level. Reprinted from Rovee-Collier et al. (1995), with permission from Elsevier.

Rovee-Collier et al. (1995) illustrated the time window concept by employing a crib mobile paradigm similar to the one described above. In this paradigm, 3-month-old infants completed two 15-min sessions with a space of 1, 2, 3, or 4 days between sessions. Retention was then tested 8 days after the first session and the results are illustrated in **Figure 1**. Infants whose second session occurred 2 days after the first session had perfect recall in the retention test because their second session was late but still within their time windows. In contrast, infants whose second session was 4 days after their first returned to their baseline level of performance because the second session occurred outside of their time windows. Finally, infants whose second session was 1 day or 3 days after the first session showed intermediate retention. For the 1-day space this was because the second session was early in the time window, whereas for the 3-day space it was because some infants could retrieve the memory whereas others could not (i.e., outside of the time window for some infants but just within the time window for others). The infants who failed to retrieve the memory did not benefit from the second session and performed close to baseline at the retention session, while those whose retrieval was successful retained the memory, suggesting that the optimal space is just before a memory is forgotten. Overall, these results show that there is an inverted-U relationship for spacing and retention in infants.

Interestingly, another study has suggested that the finding that the optimal space is just before the memory is forgotten may not be the full story. Hsu (2010) examined how long 6-, 9-, 12-, 15-, and 18-month-old infants retained a memory for an operant task, equivalent to the mobile paradigm, when their second session was completed near the end of their time window. Comparing her data with an earlier study that used the same methodology and completed the second session 24 h after the first (Hartshorn et al., 1998a), Hsu (2010) concluded that for 6-month-old infants completing the second session near the end of the time window resulted in better retention, but for the 9- to 18-month-old infants completing the second session near the end of the time window lead to worse retention than a 24 h space. It is important to note that the 9- to 18-month-old infants successfully retrieved their memory in the second session; thus if Hsu’s (2010) conclusions are correct this calls into question the assumption that more difficult retrievals are always better as suggested by some accounts of the spacing effect (e.g., Bjork and Bjork, 1992; Delaney et al., 2010). However, given the use of a between-study comparison, it would be desirable for Hsu’s (2010) finding to be replicated.

## Language and Verbal Tasks

### Adults

Unlike skill-related tasks, for language tasks spacing leads to equal or worse learning but enhanced retention. While the finding of no spacing effect for learning in language-related tasks may seem unusual, a close reading of the studies referenced reveals that this finding is very consistent (Keppel, 1964, 1967; Bahrick, 1979; Glenberg and Lehmann, 1980; Bloom and Shuell, 1981; Bahrick et al., 1993; Moss, 1995; Cepeda et al., 2006, 2009; Simone et al., 2013; Suzuki and DeKeyser, 2015). For example, Bloom and Shuell (1981) compared a spaced group that studied French words on 3 consecutive days to a massed group that studied French words all in the same day. On an immediate test conducted to assess learning, the spaced and massed groups were equivalent, but on a retention test 4 days later the spaced group’s performance was superior to the massed group; thus spacing led to equivalent learning but enhanced retention. In contrast, for a skill-related task, Shea et al. (2000) compared a spaced group that practiced a discrete timing task on 3 consecutive days to a massed group that completed the same amount of practice within the same day and found that the spaced group performed better at the end of training. The possible reasons for spacing enhancing learning in skill-related tasks but not language-related tasks will be discussed in later sections.

**FIGURE 2:**
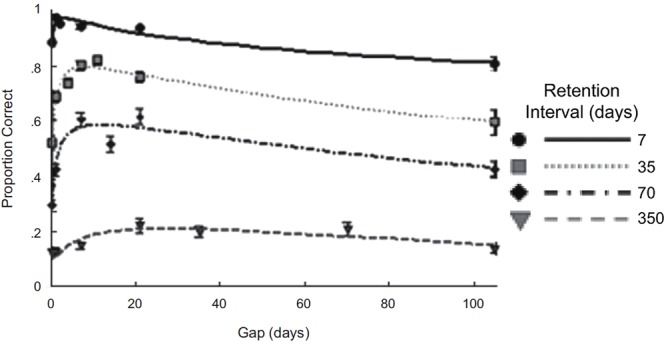
Adults’ retention performance on 32 facts for varying spacing and retention intervals. Note that performance initially improves with spacing and then declines, forming an inverted-U relationship, and overall performance drops for longer retention delays. Reprinted from Cepeda et al. (2008), with permission from Sage Journals.

For retention, similar to Rovee-Collier et al.’s (1995) findings with infants, an inverted-U curve for the spacing effect has been reported. For example, Cepeda et al. (2008) had participants study 32 facts across two sessions and then conducted a retention test. In the first session, the facts were studied and tested until each fact was correctly recalled and in the second session facts were tested twice with feedback. The spacing interval between the first and second session varied across participants, ranging from 0 to 105 days. Similarly, the delay between the second session and retention test ranged between 7 and 350 days. Initially, retention improved as the spacing interval increased but then declined, forming an inverted-U curve (see **Figure 2**). Additionally, the optimal space varied depending on the retention delay, with the optimal space being longer for longer retention delays (e.g., for the 7-day retention delay the optimal space was 3 days and for the 35-day retention delay the optimal space was 8 days).

Other studies have not used as many spacing and retention delays as Cepeda et al. (2008), but the finding that the optimal spacing interval changes depending on the retention delay has been reported for re-reading texts (Rawson and Kintsch, 2005; Verkoeijen et al., 2008; Rawson, 2012), word-pairs (Bahrick and Phelphs, 1987; Küpper-Tetzel and Erdfelder, 2012; Küpper-Tetzel et al., 2014b; Gerbier et al., 2015) and for remembering vocabulary (Küpper-Tetzel et al., 2014a). One interesting question yet to be directly addressed is what effect increasing the number of re-learning sessions has on the inverted-U curve. From the studies published to date, we hypothesize that as the number of sessions increases, the number of spacing intervals that could be considered optimal or close to optimal for a particular retention delay increases, reflecting a widening of the inverted-U curve. For example, Küpper-Tetzel et al. (2014a) used 1 relearning session and found for a 7-day delay, a 1-day spacing interval produced recall of about 86%, whereas a 10-day spacing interval produced recall of around 62%. In contrast, Bird (2010) used 4 relearning sessions and found for a 7-day delay, a 3-day spacing interval produced retention of 83.1% and a 14-day spacing interval produced retention of 80.9% (i.e., not significantly different from the 3-day spacing group).

### Infants and Children

While adults do not learn more with spaced presentations in language tasks, children do seem to learn more from spaced presentations. For example, Ambridge et al. (2006) exposed 4-year-old children to 10 sentences containing a grammatical construction they had not yet learned. The exposures were massed all in a single session or spaced across 5 consecutive days. Children in the spaced group showed much better learning than those in the massed group on a test immediately after their last training trial. In contrast, Miles (2014) had adult Korean students learn English grammar and mass their learning into 1 day or space their learning across multiple days separated by varying delays and found that on the immediate test the spaced and massed groups’ learning was approximately equal. Thus in a very similar design spacing seems to enhance learning in children but not in adults. Another way of thinking about this finding is the idea that at very short retention intervals massing is better than or equal to spacing (Cepeda et al., 2006; Maddox, 2016) is true for adults but not for children.

Additionally, in children’s language tasks, manipulating the intensity of training parallels the findings for adults learning skills. Schwartz and Terrell (1983) found that children learned more words when presentations were spread over 10 days compared to 5 days. Similarly, Childers and Tomasello (2002) found that children learned to produce more words when presentations were distributed over 4 consecutive days rather than 2 consecutive days. Childers and Tomasello (2002) also found that when children’s learning occurred across 4 sessions there was no difference in learning between groups who had an intersession interval of 24 h or 3 days, somewhat contradicting the spacing effect. There are several possible explanations for this finding. The first is that the absolute time between repetitions is not particularly important, but it is necessary for the child to have a period of sleep between each session before additional learning can occur. The second explanation is that the 3-day interval does enhance learning, but its benefits are undermined by greater forgetting, which leads to performance equivalent to the 24-h intersession interval.

Other studies have found spacing enhances children’s retention in language-related tasks (Moinzadeh et al., 2008; Sobel et al., 2011; Goossens et al., 2012). One study conducted with children is particularly interesting for its practical implications. Moinzadeh et al. (2008) compared five groups of 12- to 13-year-olds learning English as a foreign language. All of the groups completed 6 sessions: one group completed 2 sessions per day, a second group completed 1 session per day, a third group completed 1 session every alternate day, a fourth group completed 2 sessions per week and a fifth group completed 1 session per week. Learning was assessed via a test conducted 1 day after the final learning session and retention was assessed via a test conducted 1 month after the final learning session. Moinzadeh et al. (2008) reported that the group with 1 session per day performed the best on the learning test and the group with 1 session every alternate day performed the best on the retention test. This suggests that when considering the optimal spacing schedule you should consider how regularly the language will be used. For example, suppose a 12-year-old child is moving to a foreign country permanently, this study suggests that it would be optimal for them to learn with daily sessions; if however, the child was going to a foreign country for a holiday and most likely would return for multiple holidays across their lifetime, then it would be optimal for them to learn with sessions on alternate days.

## Generalization

### Adults

Interestingly, a few studies have found that spacing not only benefits the learning and retention of specific items but improves the generalization of learning. Hagman (1980) had participants learn and practice electrical testing on the same equipment or different equipment, with practice massed all in 1 day or spaced on 3 consecutive days. On a transfer test after a 2-week delay, spaced practice on different equipment resulted in better transfer than spaced practice on the same equipment. Spaced practice on the same equipment resulted in better performance on the transfer test than massed practice on the same or different equipment. Moreover, massed practice on the same or different equipment resulted in equivalent performance on the transfer test, indicating that spacing was necessary for training variations to promote generalization. Similarly, Moulton et al. (2006) compared massed and spaced groups who practiced microsurgical skills on PVC-artery models and arteries from a turkey thigh, and tested to what extent their skills transferred to a live rat 1 month after training. Moulton et al. (2006) found that the spaced group performed significantly better on a variety of outcome measures than the massed group. There is one other study that claims to show transfer for diagnostic skills, but because it used a within-subjects design and spaced training was always completed before massed training it is not possible to know whether this was due to experience alone or the spacing effect (Kerfoot et al., 2010).

The two studies described above are the only studies we are aware of that systematically examine the effect of long spacing intervals on generalization in adults, so obviously more research is needed on this aspect of the spacing effect. However, based on these studies and the studies on children discussed below, it seems probable that the spacing effect will enhance generalization in other circumstances in adults.

### Infants and Children

Studies with children have investigated the impact of spacing on generalization using a greater range of spacing intervals relative to the adult literature. For example, Vlach and Sandhofer (2012) investigated the impact of spacing on the generalization of simple and complex science concepts in 5- to 7-year-olds. The children in their study completed 4 lessons on biomes, with each lesson involving a different context (desert, grasslands, artic, ocean or swamp), and a post-test 1 week after the last lesson. The massed group completed all four lessons in 1 day, the intermediate group completed 2 lessons per day for 2 days, and the Spaced Group completed 1 lesson per day for 4 days. For simple generalization, the spaced group showed significantly greater improvement from the pre- to post-test than the massed group, and the intermediate group’s improvement was not significantly different when compared to the massed or spaced groups. In contrast, for complex generalization, the spaced group’s improvement was significantly better than both the massed and intermediate groups. In fact, the data suggest that the spaced group is the only group to show an improvement in their gain scores as the questions moved from simple to complex, though unfortunately this trend was not tested for statistical significance. Spacing therefore may provide a greater benefit for more complex generalizations.

Gluckman et al. (2014) replicated Vlach and Sandhofer’s (2012) findings, but in the post-test they included questions on the children’s memory for facts and concepts talked about during the lessons (e.g., what is a biome?), in addition to generalization questions. The spaced group showed significantly greater improvement than the massed group for simple and complex generalization questions and for memory questions. The reported means displayed the same pattern as above, with spacing benefiting complex generalizations more than simple generalizations. Gluckman et al. (2014) also tested for correlations between the memory test and generalization, hypothesizing that there would be a positive correlation between memory scores and generalization scores if generalization was related to memory for the lessons. They found no significant correlations, suggesting that in this task memory and generalization may be independent learning processes. Consistent with this finding, Wang et al. (2014) trained children on working memory games and found no effect of spacing on game performance, but they did find that spacing improved transfer performance on Raven’s Progressive Matrices test.

In contrast to the impact of spacing on learning and retention, there has been relatively little research exploring the impact of spacing over long intervals on generalization. However, the studies conducted to date allow us to tentatively conclude that greater spacing (e.g., spreading learning across 4 days vs. 2 days) seems to provide a larger benefit to generalization and that more complex generalizations seem to benefit more from spacing, independent of other more specific forms of learning. Since generalization and transfer are a very valuable part of learning it would be worthwhile for future research to examine whether these tentative conclusions are reliable and examine the extent to which spacing promotes generalization for a greater variety of tasks.

## Can Existing Theories Account for the Spacing Effect Over Long Timescales?

Currently, there seem to be two predominant types of theories for explaining the spacing effect. The first is contextual or encoding variability theories (Estes, 1955; Glenberg, 1979; Raaijmakers, 2003; Pashler et al., 2009; Maddox, 2016). These theories suggest that spaced repetitions lead to a greater variety of contextual elements being integrated into a memory than massed repetitions, and a greater variety of contextual elements means that the memory is more likely to be recalled after a delay period. Modern contextual variability theories also have a study-phase retrieval component, whereby the original memory or experience must be recalled during the repetition to integrate additional contextual elements and therefore benefit from the repetition (Raaijmakers, 2003; Pashler et al., 2009; Maddox, 2016). Furthermore, these theories explain the inverted-U curve of the spacing effect by suggesting that recall is based on the match between the test context and the contextual elements integrated during the first presentation and repetitions.

Contextual variability theories work well for explaining the data in verbal studies in adults. Test performance is based on the overlap between contextual elements stored in the memory and the contextual elements present during the test. Performance on an immediate test is often better for massed repetitions because the contextual elements at the test will be very similar to contextual elements integrated during the initial presentation and the repetition, leading to a large overlap (Delaney et al., 2010; Maddox, 2016). In contrast, for spaced repetitions the contextual elements present for an immediate test will be similar to the repetition but quite different to the first presentation. On a delayed test the contextual elements will be different to the contextual elements of the first presentation and repetition, therefore it is valuable to have a variety of contextual elements integrated into the memory so there is sufficient overlap; in this case spaced repetitions lead to a greater variety of contextual elements being integrated into the memory than massed repetitions and thus produce better retention (Delaney et al., 2010; Maddox, 2016).

However, when we look outside of the verbal data in adults, contextual variability theories have problems explaining the data. In our review, we found that spaced repetitions lead to better performance on an immediate test than massed repetitions on verbal learning tasks in young children and skill tasks in adults. According to the contextual variability theories we should observe the reverse: for an immediate test, massed repetitions should lead to better test performance than spaced repetitions due to massed repetitions resulting in a greater overlap between the test’s contextual elements and the contextual elements stored as part of the memory.

A second major class of theories explain the spacing effect in terms of retrieval difficulty (Bjork and Bjork, 1992; Benjamin and Tullis, 2010). In particular, these theories suggest that greater forgetting occurs for spaced repetitions and this makes retrieval more difficult, leading to a greater enhancement in the memory (Bjork and Bjork, 1992). Retrieval difficulty theories are supported by a number of studies of verbal memory using short spacing intervals (Bjork and Allen, 1970; Hintzman, 1974; Crowder, 1976; Benjamin and Tullis, 2010). Consistent with retrieval being more difficult, many studies of the spacing effect in language-related tasks observe that at the time of the repetition performance is worse for the spaced group than the massed group (Bjork and Allen, 1970; Glenberg, 1976; Cepeda et al., 2009). In contrast, in many of the skill-related studies using long spacing intervals the opposite is observed: at the time of the repetition performance is better for the spaced group than the massed group, suggesting that retrieval of spaced repetitions is easier than retrieval of massed repetitions (e.g., Shea et al., 2000; Dail and Christina, 2004; Molloy et al., 2012). Moreover, one study compared the retention performance of Swahili word-pairs with a spaced group that slept during their spacing interval to a spaced group that remained awake during their spacing interval and found that the sleep group performed better at the repetition and subsequently showed better performance on the retention test (Bell et al., 2014). These studies suggest that retrieval difficulty theories may not be able to account for the spacing effect over long timescales.

Another finding noted above was that the spacing effect occurred for perceptual discrimination tasks (Wright and Sabin, 2007; Molloy et al., 2012). Since the discrimination response for each trial is based on stimuli presented in close succession, it seems unlikely retrieval difficulty is influenced by the spacing of repetitions as it potentially is in verbal tasks. Similarly, it seems unlikely that stored contextual elements play a significant role in the ability to make discriminations in these tasks. Significantly, however, for the theory proposed below, memory consolidation plays a critical role in improving participants’ discrimination skills (Gais et al., 2000; Atienza et al., 2004; Gaab et al., 2004).

## A Reconsolidation Account of the Spacing Effect

### Reconsolidation as a Mechanism

In the past there have been attempts to explain the spacing effect in terms of memory consolidation (Landauer, 1969; Wickelgren, 1972). However, these theories were generally rejected because of theoretical and empirical issues (Bjork and Allen, 1970; Hintzman, 1974; Dempster, 1989; Delaney et al., 2010). In the decades since these papers were published there have been many significant developments in our understanding of consolidation and these developments are what make a reconsolidation account a viable hypothesis for explaining the spacing effect over long timescales.

When the earlier consolidation theories of the spacing effect were published the concept of memory reconsolidation was not widely studied or adopted (Nader and Hardt, 2009; Sara, 2010). Instead, the dominant perspective was that a memory was initially unstable and then over time consolidated in a linear manner (Nader and Hardt, 2009; Sara, 2010). A resurgence of interest in memory reconsolidation led to experiments showing that this perspective was partially incorrect (Nader and Hardt, 2009): after the initial consolidation period when a memory was retrieved it returned to being unstable and sensitive to disruption. This period of instability probably provides a net benefit, as it is necessary for additional experiences to modify and build on the pre-existing memory trace (Alberini, 2011).

One of the ways researchers gained a better understanding of consolidation and reconsolidation was through experiments that used protein synthesis inhibitors such as anisomycin (Nader et al., 2000; Suzuki et al., 2004; Nader and Hardt, 2009; Wang et al., 2009). The initial consolidation experiments injected a protein synthesis inhibitor a little before or after training and found that memory was generally unaffected 0–2 h after training, but when the memory was tested 24 h after training it was disrupted (Davis and Squire, 1984; Goelet et al., 1986; Meiri and Rosenblum, 1998; Schafe et al., 1999; McGaugh, 2000). Thus a short-term memory could be sustained for a few hours without generating new proteins but new proteins were needed for a long-term memory. Later, when research on reconsolidation developed, similar findings were observed (Nader et al., 2000; Schafe and LeDoux, 2000; Suzuki et al., 2004; Rossato et al., 2007). Researchers discovered that when the memory was retrieved, injecting protein synthesis inhibitors into brain areas associated with the memory disrupted the memory after 24 h but not when tested a few hours after training. These findings provide several important pieces of information about consolidation and reconsolidation. Firstly, the neural consolidation processes which influence the development of the long-term memory take time to develop and may not affect the memory over the first few hours after the initial training or reactivation. Secondly, the memory gets additional consolidation (reconsolidation) from the reactivation.

Other experiments using protein synthesis inhibitors and a variety of other techniques have further developed our understanding of memory reconsolidation. Researchers have determined two functions for memory reconsolidation: altering an existing memory trace in response to new experiences and strengthening a memory (Lee, 2008; Alberini, 2011; Inda et al., 2011). On a behavioral level, memory strengthening has been identified as improved learning and better retention (Morris et al., 2006; Lee, 2008; Inda et al., 2011). Additionally, research has shown that memory reconsolidation seems to be a basic memory process occurring across many different tasks and species (Walker et al., 2003; Pedreira et al., 2004; Alberini, 2005; Forcato et al., 2007). In particular, it has been demonstrated in humans using both motor skill and verbal tasks (Walker et al., 2003; Forcato et al., 2007, 2009; Coccoz et al., 2011; De Beukelaar et al., 2014). Based on this research, we can be confident that the reconsolidation process is playing a role in the experiments described earlier. However, the critical question is: does the time between repetitions influence the degree to which consolidation and reconsolidation strengthen and improve memories? Or alternatively, is reconsolidation’s effect on memory independent of the timing of repetitions and merely mediates another mechanism which is responsible for the spacing effect? For example, it might be the case that the reconsolidation process is responsible for integrating additional contextual elements into a memory and it is the addition of these elements which produce the spacing effect. Before we answer this question directly, it is worthwhile to address another development in our understanding of the consolidation of memory.

A second development in memory consolidation research is a much better understanding of the significance of sleep (Stickgold, 2006). Sleep plays an active role in memory consolidation. During sleep memories are reactivated (Pavlides and Winson, 1989; Wilson and McNaughton, 1994; Ji and Wilson, 2007; Oudiette and Paller, 2013) and the different stages of sleep are associated with different tasks and aspects of performance, suggesting that sleep-based consolidation makes a qualitatively different contribution to memory than the waking state (Gais et al., 2002; Walker and Stickgold, 2004; Marshall et al., 2006; Stickgold, 2006). On a behavioral level, there are parallels between the sleep literature and the spacing effect studies we reviewed earlier. In skill learning tasks, a period of sleep leads to better performance with no additional practice (Walker et al., 2003; Kuriyama et al., 2004; Fischer and Born, 2009) and in verbal tasks, sleep generally reduces forgetting but does not improve performance (Drosopoulos et al., 2007; Lahl et al., 2008; Abel and Bäuml, 2014). Additionally, sleep is important for the generalization of memories (Stickgold and Walker, 2013). Similar to the reconsolidation literature, studies investigating sleep and learning indicate that consolidation during sleep is influencing the same dependent variables (learning and retention) as the spacing effect and sleep, like the spacing effect, requires time to influence these variables. Thus we return to the question does the spacing of repetitions influence the benefit gained by sleep-consolidation and reconsolidation? Or are the benefits from sleep-consolidation and reconsolidation independent of the spacing of repetitions?

Logically, it seems likely that the spacing of repetitions would influence the consolidation and reconsolidation processes and their beneficial effects on learning and retention. There are multiple studies indicating that consolidation during the night is influenced by an individual’s learning experiences during the day (Pavlides and Winson, 1989; Maquet et al., 2000; Poe et al., 2000; Laureys et al., 2001). For example, Gais et al. (2002) found that participants who learned word-pairs showed a greater density of sleep spindles on the following night than participants who completed a word task that did not require long-term memory. It seems likely, therefore, that the spacing of repetitions over different numbers of days might influence memory consolidation during sleep. For example, in the Arthur Jr. et al. (2010) study reviewed earlier, the spaced group learned the naval command simulation over 2 weeks and the massed group learned it over 1 week. It seems probable that the spaced group might get a greater degree and quality of reprocessing during sleep than the massed group.

There is also some experimental evidence that suggests that the spacing of repetitions influences consolidation and reconsolidation. Before we look at this evidence, it is worthwhile to state more clearly what the reconsolidation account of the spacing effect entails. Essentially, we are suggesting that greater time between repetitions provides more time for the memory to consolidate and this greater degree of consolidation makes the additional consolidation (reconsolidation) induced by a repetition more effective at enhancing the memory. Furthermore, part of the reconsolidation process is further processing of the memory during sleep.

Many of the verbal and skill tasks reviewed earlier in this paper used a design where the massed group completed all of their trials in 1 day and the spaced group completed their trials across 2 days. One study that investigated memory reconsolidation had a similar set-up. In this study, rats completed two trials of context-shock conditioning (Lee, 2008). Some of these rats completed the two trials all in a single day and others completed the trials across 2 days and the memory of both groups was tested on the third day. After their second trial the rats were either injected with a substance that inhibited brain derived neurotrophic factor (BDNF), a protein that has a variety of functions related to neuron growth and neural plasticity, or a substance that inhibited ZIF268, a transcription factor involved in learning and memory processes. The researchers found that when the second trial occurred on the first day, inhibiting BDNF expression disrupted the memory but inhibiting ZIF268 expression had no effect. In contrast, when the second trial occurred on the second day, inhibiting BDNF had no effect but inhibiting the expression of ZIF268 disrupted the memory. Furthermore, if no reactivation trial occurred on the second day, inhibiting ZIF268 had no effect on memory performance. The findings of this study suggest that a repetition engages slightly different neural mechanisms depending on the spacing of the repetition. In particular, our interpretation of this study is that ZIF268 is particularly important only on day 2 because it is part of the process building on the consolidation that occurred the previous night. Consistent with this perspective, other researchers have also found that some of the mechanisms used by memory reconsolidation are different to the initial consolidation process (Taubenfeld et al., 2001; Bozon et al., 2003; Bahar et al., 2004).

In mammals some memories shift from being dependent on the hippocampus to being dependent on the cortex. This process is generally called systems consolidation and is thought to be beneficial for long-term memory (Milner, 1989; Alvarez and Squire, 1994). If spacing repetitions allows more time for consolidation and this consolidation makes the subsequent reconsolidation process more effective, this should lead to a greater degree of systems consolidation. Lehmann et al. (2009) conducted a study where one group of rats received 12 context-shock trials spread across 6 days (i.e., spaced rats) and another group received 12 context-shock trials all in 1 day (i.e., massed rats). The rats then had their hippocampus lesioned 7–10 days after their initial learning session. After lesioning, the spaced rats continued to show fear to the context whereas the massed rats were amnesiac, demonstrating that the spacing of repetitions increased the rate of systems consolidation such that the memory was hippocampal-independent in spaced rats but hippocampal-dependent in massed rats. This study therefore provides some initial evidence that spaced repetitions enhance the consolidation of memories to a greater extent than massed repetitions.

The relationship between the spacing of repetitions and memory consolidation was also explored in a study by Vilberg and Davachi (2013) using a within-subjects design. Participants studied and restudied word-object pairs and word-scene pairs. Massed pairs were restudied after 20 min, while spaced pairs were restudied after 24 h, and memory for all of the pairs was tested 24 h after the restudy period. While the participants were restudying the pairs they were scanned in an fMRI machine. Spaced word-object pairs remembered on the test showed a greater connectivity between the hippocampus and the perirhinal cortex than massed word-object pairs that were remembered. Additionally, the likelihood of spaced word-object pairs being forgotten could be predicted by the connectivity between the hippocampus and the perirhinal cortex, but the same prediction could not be made for the massed word-object pairs. The results for word-object pairs are consistent with the proposal that allowing time for consolidation enables spaced repetition to be more effective than the massed repetition. No relationships were identified for the word-scene pairs, but this may have been due to these pairs consolidating differently and the measures used were unable to detect their consolidation.

Additional evidence that reconsolidation is more effective for partially consolidated memories comes from a study by Tse et al. (2007). In their study, rats learned the spatial arrangement of an arena and that a flavored pellet in the start box meant that food corresponding to that flavor was hidden in a specific sand-well in the arena (there were six flavors and six sand-wells). After the initial task was learned over several weeks and consolidated, Tse et al. (2007) introduced two new sand-wells into the arena that were associated with new flavors. Learning these new flavor-location associations would have induced reconsolidation as the memory for the arena would be modified to integrate the new learning. The two new flavor-location associations were learned after a single trial and were independent of the hippocampus 48 h later. Normally, new associations take several weeks to become independent of the hippocampus, suggesting that the framework the rats had established greatly increased the rate of memory consolidation. Additionally, reinforcing their findings, Tse et al. (2011) found that learning new associates within a previously established arena led to a greater expression of genes associated with plasticity in neocortical areas than learning new associates in a new arena or retrieving previously learned associates. Like the other studies reported on, Tse et al.’s (2007, 2011) results support the central principle of the reconsolidation account of the spacing effect: that allowing memories time to consolidate enhances the reconsolidation of memories. Tse et al.’s (2007, 2011) studies were designed to gain a better understanding of the schema effect, which is the observation that establishing a framework of knowledge facilitates memory for additional learning that can be fit within the same framework. The spacing effect over long timescales is likely to partially overlap with the schema effect, the core difference being that with the spacing effect, the additional learning has a much higher degree of similarity to the established framework.

From our review of the evidence relating reconsolidation to the spacing effect, we can establish why spaced repetitions might be beneficial for learning and retention. For learning, spacing enables some initial learning to consolidate and then at the repetition the reconsolidation process can more effectively integrate and consolidate additional learning, thus building on the earlier consolidation process. For retention, spacing enables the memory to consolidate and then the subsequent reconsolidation process is more effective at enhancing the memory, making it more durable.

### Reconsolidation and the Inverted-U Curve of the Spacing Effect

We have discussed why a longer spacing interval produces better memory than a shorter interval. However, as we discussed earlier, longer spacing intervals are not always better than shorter intervals and there is an inverted-U curve for the spacing interval and its effect on retention. It is worth comparing the inverted-U curve that was found for infants and for adults. For adults, the inverted-U curve shifted depending on the length of the retention delay, with the optimal space being longer for longer retention delays; for infants (Rovee-Collier et al., 1995; Galluccio and Rovee-Collier, 2006), changing the retention delay for the same set of spaces was not directly tested, but for 3-month-old infants, going beyond the optimal spacing interval led to the infant performing at baseline, which means the optimal interval could not have shifted with a longer retention delay. We think that the inverted-U curve for both adults and children can be accounted for by assuming that forgetting reduces the effectiveness of memory reconsolidation.

The data for 3-month-old infants’ retention of the crib mobile paradigm is relatively simple to explain (Rovee-Collier et al., 1995). The 2-day spacing interval produced the best retention (**Figure 1**) because retrieval is successful in all or almost all of the infants and sufficient time has passed to enable the reconsolidation process to be quite effective. For the 3-day interval, some of the children retrieved the memory initiating reconsolidation and others did not, leading to a return to baseline performance on the retention test. The intermediate level of retention is a result of averaging across these two sub-groups. For the 4-day interval the majority of the children can no longer retrieve the memory, thus reconsolidation does not occur, leading to very poor retention.

We can use the same principles to understand the inverted-U curve observed by Cepeda et al. (2008) (**Figure 2**). It is important to note that the infants’ retention was based on performance on a single task whereas the adults’ retention was based on the recall of 32 facts and this difference most likely leads to the observed differences in the inverted-U curves. It seems a reasonable assumption that amongst the 32 facts there were differences in strength based on factors such as the attention given to that fact during learning or the memorability of the fact. These differences in strength might result in a slightly different forgetting curve for each fact and a different point in time when it is optimal to repeat each fact. The optimal spacing interval for a particular retention delay across all of the facts is the one long enough to provide a substantial benefit to some facts but short enough so that not too many facts are so weak that they receive little or no benefit from the repetition. The optimal spacing interval and other points on the curve shift depending on the retention delay, because as the retention delay increases, the facts need more time before the repetition will be beneficial enough to be recalled after the longer, more difficult delay. However, as a consequence of the repetition occurring later, some weaker facts that would have benefited from an earlier repetition receive little or no benefit from the repetition, but these weaker facts would have been forgotten across the longer retention delay anyway. For example, for a retention delay of 7 days the optimal spacing interval is around 3 days. Hypothetically this could mean that 98% of the facts benefit from the repetition and 2% of the facts are too weak to benefit and this results in 94% of the facts being recalled in the retention test. For a retention delay of 35 days, the optimal spacing interval is around 8 days. At this retention delay, a 3-day spacing interval still benefits 98% of the facts, but due to the long delay only 70% of the facts are remembered in the retention test. In contrast, for a spacing interval of 8 days, there is greater forgetting *before* the repetition, perhaps resulting in only 90% of the facts benefiting from the repetition. However, those facts which do benefit from the repetition receive a larger benefit, leading to less forgetting. Therefore at the 7-day retention delay, the 8-day spacing interval might lead to recall of 88%, which is worse than the recall of 94% produced by the 3-day spacing interval; but at the 35-day retention delay, the 8-day spacing interval might result in recall of 80%, which is better than the 70% recall at the 3-day spacing interval. Thus by taking into account that multiple facts are learned and that there are probably differences in the benefits that the facts receive from repetitions, we can account for the core characteristics of the inverted-U curve for retention.

### Accounting for the Effects of Spacing on Learning

One of the problems with some of the existing theories of the spacing effect we identified above was that they are unable to explain why under some circumstances spacing benefits learning and retention, while in others it benefits retention but does not enhance learning. One finding is that the spacing effect enhances learning in language-related tasks in children but not in adults. Noticeably, the adults can learn a lot of words or word pairs within a single session. For example, adults can learn 40 new word-pairs in the first session (e.g., Bahrick and Hall, 2005; Cepeda et al., 2009). In contrast, children’s ability to learn new words is more limited, with spacing studies teaching children 6–16 new words, which are not all remembered even after multiple learning sessions (Schwartz and Terrell, 1983; Childers and Tomasello, 2002). Part of the reason the spacing effect occurs for children and not adults in this context might be that children require time for consolidation between presentations, whereas adults’ rapid learning makes consolidation unnecessary for acquisition, but they forget the words relatively quickly and do require consolidation and reconsolidation to make long-lasting memories.

Another finding is that adults show the spacing effect for learning skill-related tasks but not language-related tasks. A similar finding occurs in the sleep literature: after a period of sleep, performance improves in skill tasks (Mednick et al., 2003; Walker et al., 2003; Kuriyama et al., 2004; Fischer and Born, 2009; De Beukelaar et al., 2014) but generally declines in language tasks, with sleep’s beneficial effects occurring due to reduced forgetting (Yaroush et al., 1971; Drosopoulos et al., 2007; Stickgold and Walker, 2007; Lahl et al., 2008; Abel and Bäuml, 2014). Since we believe that sleep consolidation contributes to the spacing effect, the findings in the spacing literature and sleep literature can be explained in a similar way. Noticeably, in skill-learning tasks, participants are generally only learning one skill and their acquisition is gradual, occurring over days. In contrast, for language tasks, words or word-pairs are generally acquired rapidly with the difficulty of the task coming from the large number of words they have to learn. The explanation for the difference between language-related and skill-related tasks is thus essentially the same as the explanation for the difference between children and adults for language-related tasks. After a certain number of repetitions within a single day, skill-related tasks require consolidation for additional improvements in performance, whereas in language-related tasks repetitions within a single day remain effective until the word-pairs are acquired but consolidation and reconsolidation is necessary for other improvements in the memory such as reduced forgetting and resistance to interference. Why might there be differences in acquisition for adults in language tasks compared to skill tasks and language tasks in children? A plausible explanation is adults’ daily experience with language facilitates acquisition in language tasks, while adults do not have the same degree of experience in motor skill tasks and children obviously do not have the same degree of experience with language. An implication of this idea is that an adult’s expertise or experience in a particular area will impact the benefits received from the spacing effect. For example, spacing might be less beneficial for expert pianists learning a new piece than for novice pianists.

The observation that the spacing effect enhances the generalization of learning fits well with the reconsolidation account. Numerous studies have demonstrated that one of the important functions of consolidation is the generalization of learning (Fischer et al., 2006; Gómez et al., 2006; Stickgold and Walker, 2013; Friedrich et al., 2015), so if the reconsolidation induced by spaced repetitions enhances the consolidation processes (i.e., as reported for systems consolidation earlier in the study by Lehmann and McNamara, 2011), you would expect generalization to be enhanced as well. Additionally, the finding that spacing benefits complex generalizations more than simple generalizations is paralleled in the consolidation literature, where the more complex parts of a task receive the greatest benefit from memory consolidation (Kuriyama et al., 2004).

### The Reconsolidation Account in Comparison to Previous Consolidation Accounts

There are significant differences between the use of reconsolidation as a primary mechanism to explain the spacing effect and the use of consolidation as a mechanism of the spacing effect as explored in earlier consolidation accounts (Landauer, 1969; Wickelgren, 1972). An important difference is the significance placed on memory retrieval. Retrieval of the original memory trace is necessary for the reconsolidation process, which involves a period of instability and allows for modifications of the memory trace (Sara, 2000; Alberini and LeDoux, 2013). Interestingly, the development of retrieval’s significance in the reconsolidation literature is essentially the same as the concept of study-phase retrieval that has developed in the spacing effect literature. Study-phase retrieval is the observation that the original experience or memory for an item is often retrieved when a repetition occurs and it has been observed that study-phase retrieval is necessary for the spacing effect to occur (Hintzman et al., 1975; Thios and D’Agostino, 1976; Delaney et al., 2010). Study-phase retrieval is therefore an intrinsic part of a reconsolidation account of the spacing effect, while it was not part of earlier consolidation accounts.

The role that retrieval plays in modifying the memory trace leads the reconsolidation account to different predictions than previous consolidation accounts. In Landauer’s (1969) consolidation account of the spacing effect, when an item was presented twice, both presentations initiated a consolidation process and memory performance is a summation of the consolidation initiated by these two presentations. However, the consolidation initiated by the second presentation disturbs the consolidation of the first presentation. Therefore a massed repetition leads to less total consolidation and poorer memory performance than a spaced repetition because the consolidation of the first presentation is disturbed soon after it is initiated (Landauer, 1969; Hintzman, 1974). The implication of this theory is that the locus of the spacing effect is on the first presentation of an item rather than the second. Empirical evidence later indicated that the second presentation or retrieval of a memory was actually the locus of the spacing effect (Hintzman et al., 1973; Hintzman, 1974). In contrast, in a memory reconsolidation account of the spacing effect the locus of the spacing effect is on the second presentation. This part of the theory is derived generally from the fact that, in the reconsolidation literature, retrieval is acting to modify the memory trace and is supported more specifically by the Lee (2008) study described earlier. Recall, in the Lee (2008) study, when two context-shock trials occurred in a single day inhibiting BDNF after the second trial disrupted the memory but inhibiting ZIF268 did not, whereas when the two trials occurred across 2 days inhibiting BDNF had no effect but inhibiting ZIF268 disrupted the memory. For concision, we left out an additional condition Lee (2008) included, whereby the same procedure was followed except that the rat received only one context-shock trial and BDNF or ZIF268 was then inhibited. In this case, the results were identical to when two trials occurred on the same day: inhibiting BDNF disrupted the memory while inhibiting ZIF268 had no effect. This suggests that the effectiveness of the second trial was undermined by having it on the same day as the first trial.

Another difference between the reconsolidation account and the earlier consolidation accounts is the reason why delaying the repetition is beneficial. For example, Landauer (1969) emphasize that delaying the repetition is important because it increases the *amount* of consolidation. In the earlier sections, we have already reviewed evidence that the repetition engages different neural processes depending on when it occurs (e.g., see the earlier discussions of Tse et al., 2007; Lee, 2008) and that consolidation during sleep makes important qualitative changes to the memory. The reconsolidation account therefore puts greater emphasis on the idea that delaying a repetition is beneficial because of changes in the *state* of the memory induced by memory consolidation and reconsolidation. Part of these changes is re-organizing a memory trace to create a more effective representation in the brain (Stickgold and Walker, 2007). Moreover, Vilberg and Davachi’s (2013) finding that when an item has not consolidated (by forming greater cortical connections) the spaced repetition is not as beneficial, is consistent with this perspective.

Another difference between the reconsolidation account and previous consolidation accounts is the timescale of consolidation. The earlier consolidation accounts of the spacing effect suggested that consolidation influenced the memory on very short timescales, as used in the early spacing experiments [e.g., 4.5 s massed vs. 18 s spaced (Bjork and Allen, 1970)]. Part of this assumption probably stems from relying on early studies that used electroconvulsive shock (ECS) to disturb the consolidation process (Landauer, 1969). These studies showed ECS disturbed memory on short timescales. However, some researchers have argued that ECS disturbs retrieval instead of consolidation (Nielson, 1968; Miller and Marlin, 2014) and most of the recent work on consolidation and reconsolidation uses different techniques to disturb consolidation (e.g., Schafe and LeDoux, 2000; Dębiec and Ledoux, 2004; Suzuki et al., 2004; Bekinschtein et al., 2007). These studies find that a disturbance of consolidation and reconsolidation has no effect when tested seconds or hours after consolidation has been disturbed but does impact it the following day. Based on this research we believe that, while a reconsolidation account is a compelling hypothesis for explaining the spacing effect over long timescales, it cannot explain the spacing effect over short timescales. This viewpoint is also consistent with the behavioral spacing literature where the studies posing problems for consolidation and reconsolidation accounts of the spacing effect all use very short timescales (Bjork and Allen, 1970; Crowder, 1976).

If the reconsolidation account cannot explain the spacing effect over short timescales, it is a natural question to ask then why not rely on a single mechanism that can explain the spacing effect over both short and long timescales? The reason is that a combination of mechanisms seems required to explain the data collected on the spacing effect, memory consolidation and reconsolidation so far. Retrieval difficulty or contextual variability theories might be able to explain the data over short timescales, but, as discussed earlier, these theories have some difficulty with elements of the behavioral and neuroscientific data on the spacing effect across long timescales (e.g., Ambridge et al., 2006; Lehmann and McNamara, 2011; Vilberg and Davachi, 2013). For example, we mentioned earlier that with retrieval difficulty theories it is difficult to account for the fact that in motor skill tasks spaced repetitions after 24 h are generally easier than massed repetitions (Shea et al., 2000; Dail and Christina, 2004). This poses no problem for a reconsolidation account because in this account greater retrieval difficulty for the spaced group is not necessary for the spacing effect to occur. Furthermore, in a sense this finding supports the reconsolidation account because spaced repetitions are easier due to memory consolidation during sleep (Karni et al., 1994; Stickgold et al., 2000; Walker et al., 2003; Korman et al., 2007; Stickgold and Walker, 2007; Fischer and Born, 2009; Simmons, 2011; Bell et al., 2014; De Beukelaar et al., 2014).

### Testing a Reconsolidation Account of the Spacing Effect

While we think the evidence so far suggests that a reconsolidation account of the spacing effect is a compelling hypothesis, obviously further evidence needs to be collected to support or falsify it. There are a number of ways to test the reconsolidation account. One way is by examining whether variables in rats and humans that influence reconsolidation also influence the spacing effect in a similar manner. For example, increasing the strength of training generally makes memory reconsolidation more difficult to induce (Suzuki et al., 2004; Wang et al., 2009). If the spacing effect depends on memory reconsolidation, the strength of training should reduce the effect size of the spacing effect. Future research could also investigate other variables that influence memory reconsolidation, such as the method of memory reactivation and the age of the memory.

Another way to test the reconsolidation account is by examining the neural correlates of memory consolidation, such as the hippocampal to cortical shift. The reconsolidation account predicts that spaced repetitions will lead to a greater hippocampal to cortical shift than massed repetitions. This prediction can be tested through a number of different methods. One method is by examining at what time point a memory becomes independent of the hippocampus. For this test it would be worthwhile to replicate Lehmann and McNamara (2011), as well as testing other tasks and spacing intervals. A second method is by examining the expression of genes associated with neural plasticity: spaced repetitions should be associated with greater expression of genes for neocortical plasticity than massed repetitions. A third method is by using fMRI; on a later test spaced repetitions should lead to stronger connections with neocortical areas and a greater degree of reorganization than massed repetitions.

Earlier in this paper, we suggested that a greater degree of prior experience might explain the observations for the spacing effect for learning. This hypothesis can easily be tested experimentally. For example, in an experiment, adults could learn a maze through spaced repetitions or massed repetitions, after learning 10 different mazes across multiple days (experienced) or not learning 10 mazes (novice). The reconsolidation account predicts that experienced participants will benefit less from spacing for learning than novice participants.

Another implication of the reconsolidation account presented here is that there should be differences in how variables influence the spacing effect at short and long timescales. For example, there is some initial evidence that encoding/contextual variability influences the spacing effect on short timescales (Verkoeijen et al., 2004; Maddox, 2016), however, one study that has explored the spacing effect over long timescales (and as far as we are aware this is the only one) found that contextual variability was beneficial for some participants but its effects were independent of the spacing effect (Smith and Rothkopf, 1984). If the reconsolidation account is correct, this general pattern should be reliable: the influence of a mechanism that affects the spacing effect on short timescales will be reduced or disappear when the spacing effect is investigated using long timescales.

## Conclusion

By restricting our focus to the spacing effect over long timescales, considering learning and retention and potential differences between adults and children, we have highlighted some patterns in the literature not observed in past reviews. In children, spacing enhances word and grammar learning. In adults, spacing enhances the learning or acquisition of skills but does not enhance the learning of words or grammar. However, in both adults and children, spacing generally enhances the generalization of learning and the retention of words, grammar, and skills. Accounts of the spacing effect that involve contextual variability and retrieval difficulty have some difficulty in accounting for these findings but, they can be accounted for by considering participants, degree of prior experience and how that might interact with consolidation processes.

We have proposed a reconsolidation account of the spacing effect and, by examining the neuroscientific evidence related to the spacing effect, we have observed some initial evidence that supports it. The initial evidence suggests spaced repetitions engage different neurophysiological mechanisms than massed repetitions. Spaced repetitions enhance the consolidation of memories to a greater extent than massed repetitions and providing time for memories to consolidate enhances the consolidation/reconsolidation of additional learning that can be fit into the same framework, resulting in faster learning and better retention. Some kind of account of the spacing effect involving consolidation and reconsolidation seems the best way to make sense of this data. Finally, there are aspects of the behavioral data that are better accounted for by a reconsolidation account, such as the finding that spaced repetitions are often easier than massed repetitions. Therefore, based on the neuroscientific and behavioral evidence, the reconsolidation account of the spacing effect is a hypothesis worth exploring.

## Author Contributions

CS conceived the work, drafted and revised the manuscript, approved the final manuscript, and agreed to be accountable for all aspects of the work in ensuring that questions related to the accuracy or integrity of any part of the work are appropriately investigated and resolved. DS contributed to the conception of the work, revised the manuscript, approved the final manuscript, and agreed to be accountable for all aspects of the work in ensuring that questions related to the accuracy or integrity of any part of the work are appropriately investigated and resolved.

## Conflict of Interest Statement

The authors declare that the research was conducted in the absence of any commercial or financial relationships that could be construed as a potential conflict of interest.
